# Supplementary figures and images for: Combined Effect of High Hydrostatic Pressure and Proteolytic Fraction P1G10 from Vasconcellea cundinamarcensis Latex against Botrytis cinerea in Grape Juice
Source: Foods. 2023 Sep 12;12(18):3400. doi: 10.3390/foods12183400 (PMC10530099; doi:10.3390/foods12183400)

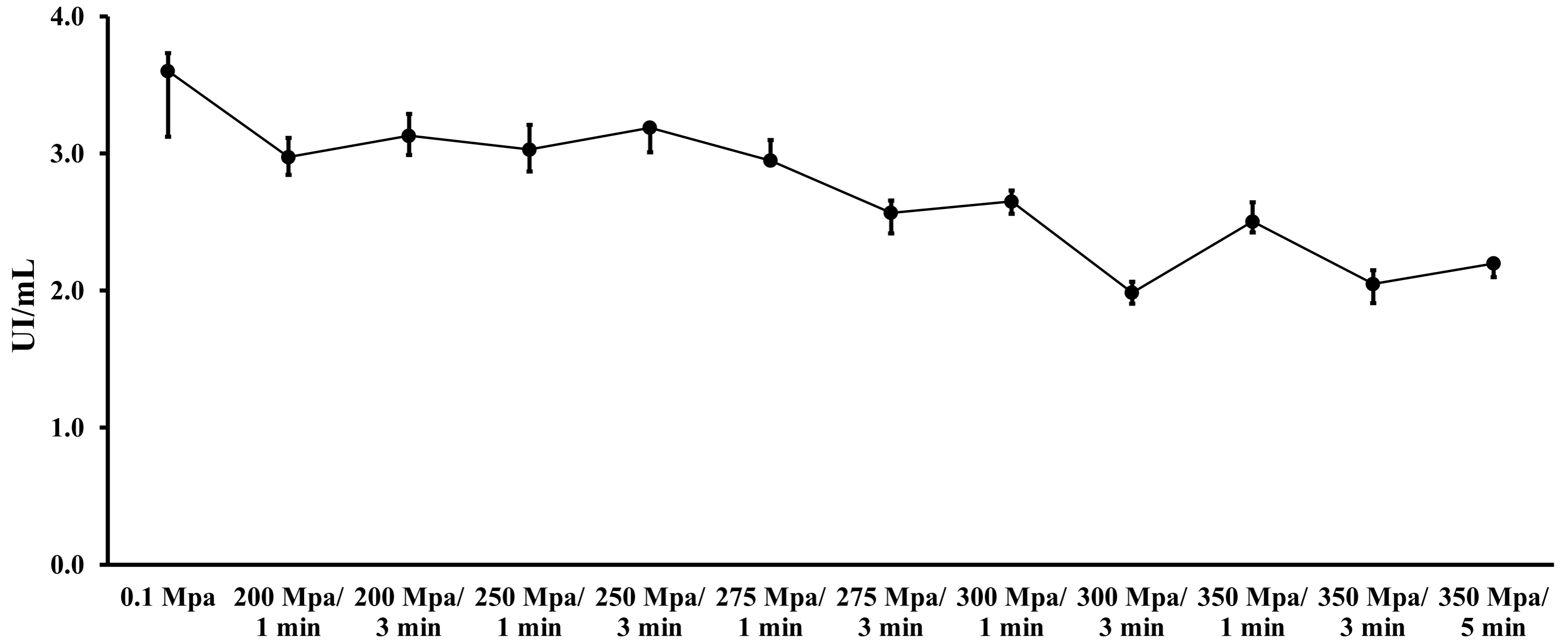

Supplement: Supplementary file 1 [file foods-12-03400-s001.zip › foods-2585227-supplementary.pdf]
